# Moose selecting for specific nutritional composition of birch places limits on food acceptability

**DOI:** 10.1002/ece3.3715

**Published:** 2017-12-20

**Authors:** Hilde K. Wam, Annika M. Felton, Caroline Stolter, Line Nybakken, Olav Hjeljord

**Affiliations:** ^1^ Division of Forestry and Forest Resources NIBIO Ås Norway; ^2^ Faculty of Forest Sciences Swedish University of Agricultural Science Alnarp Sweden; ^3^ Department of Animal Ecology and Concervation University of Hamburg Hamburg Germany; ^4^ Faculty of Environmental Sciences and Natural Resource Management Norwegian University of Life Sciences Ås Norway

**Keywords:** carrying capacity, fitness, mineral, nitrogen, preference, ungulate

## Abstract

Despite decades of intense research, it remains largely unsolved which nutritional factors underpin food selection by large herbivores in the wild. We measured nutritional composition of birch foliage (*Betula pubescens*) available to, and used by, moose (*Alces alces*) in natural settings in two neighboring regions with contrasting animal body mass. This readily available food source is a staple food item in the diet of moose in the high‐fitness region, but apparently underutilized by moose in the low‐fitness region. Available birch foliage in the two regions had similar concentrations of macronutrients (crude protein [CP], fiber fractions, and water‐soluble carbohydrates [WSC]), although a notably lower variation of WSC in the low‐fitness region. For minerals, there were several area differences: available birch foliage in the low‐fitness region had less Mg (depending on year) and P, but more Ca, Zn, Cu, and Mn. It also had higher concentrations of some plant secondary metabolites: chlorogenic acids, quercetins, and especially MeOH‐soluble condensed tannins. Despite the area differences in available foliage, we found the same nutritional composition of birch foliage used in the two regions. Compared to available birch foliage, moose consistently used birch foliage with more CP, more structural fiber (mainly hemicellulose), less WSC, higher concentrations of several minerals (Ca, Zn, K, Mn, Cu), and lower concentrations of some secondary metabolites (most importantly, MeOH‐soluble condensed tannins). Our study conceptually supports the nutrient‐balancing hypothesis for a large herbivore: within a given temporal frame, moose select for plant material that matches a specific nutritional composition. As our data illustrate, different moose populations may select for the same composition even when the nutritional composition available in a given food source varies between their living areas. Such fastidiousness limits the proportion of available food that is acceptable to the animal and has bearings on our understanding and application of the concept of carrying capacity.

## 1 INTRODUCTION

Foraging decisions are complex trade‐offs, particularly for wide‐roaming and long‐living species like large herbivores (Parker, Barboza, & Gillingham, 2009). A fitting quote is that these animals use “most of the best and least of the worst but some of everything” (Langvatn & Hanley, 1993, p. 168). Accumulated knowledge from the field of nutritional ecology shows with increasingly detail how animal metabolism and food selection comprise sets of synergetic or antagonistic assimilation and allocation pathways of food constituents (e.g., Boggs, 2009; Felton, Felton, Lindenmayer, & Foley, 2009; Felton et al., 2016; Raubenheimer, Simpson, & Mayntz, 2009; Sperfeld, Martin‐Creuzburg, & Wacker, 2012). So far, integrated study approaches which consider nutritional components in synchrony are comparatively rare for large herbivores in natural settings (e.g., Beck & Peek, 2005; Shipley, Blomquist, & Danell, 1998; Tixier et al., 1997; Vangilder, Torgerson, & Porath, 1982).

Behind all animals' food selection lies the need to assimilate adequate quantities of energy and various nutrients from the environment. The challenge is that these components are only available in sets embedded in a food item (as “food packages”), while each component has its own functional implications for the animal. Some components are necessary for maintaining life, while others are dangerous and should be avoided (like toxins used by plants to defer herbivory, Freeland & Janzen, 1974). However, some necessary nutrients can be harmful if ingested in excessive amounts, and some toxins are beneficial to the consumer in low quantities (Raubenheimer & Simpson, 2009). To complicate matters further, food components have interactive effects (Björndal, 1991). For example, if the food contains high levels of carbohydrates relative to protein, then the animal's ability to avoid a carbohydrate overdose depends on its capacity to endure a protein shortage. Likewise, high fiber intake may inhibit mineral absorption (Freeland‐Graves, Sanjeevi, & Lee, 2015), while the intake of several macronutrients can influence the effects of toxins (Simpson & Raubenheimer, 2012).

Achieving nutritional homeostasis, therefore, involves a complex interplay between variable foods, and multiple and changing needs as the animal goes through different life stages and seasons. While evolution has equipped animals with mechanisms to deal with these complexities (Behmer, 2009), nutritional ecologists are still puzzled to understand them. One major explanation is that the nutritional value and selection of a given food item may show extensive spatiotemporal variation (Morgantini & Hudson, 1989). Designing studies that grasp most of the variance of interest can therefore be difficult without extensive prior knowledge of the study system. For example, Jones, Strickland, et al. (2010) found that the availability of various soil resources providing different nutritional planes for deer can explain as much as 78% of the variation in their body mass. McArt et al. (2009) measured protein availability in major browse species for moose and found that the within‐species variation between two areas was so large that a similar diet and food intake would yield a substantially different protein balance for the moose. These relationships inferred at the level of nutrient availability appear much stronger than those typically inferred at the level of food availability (e.g., Herfindal et al., 2013; Wam, Hjeljord, & Solberg, 2010). To better elucidate the potentially masked and masking factors in food–fitness relationships, researchers need to address its finer print, that is, the nutritional underpinnings driving the animals' food choices (Parker et al., 2009).

**Figure 1:**
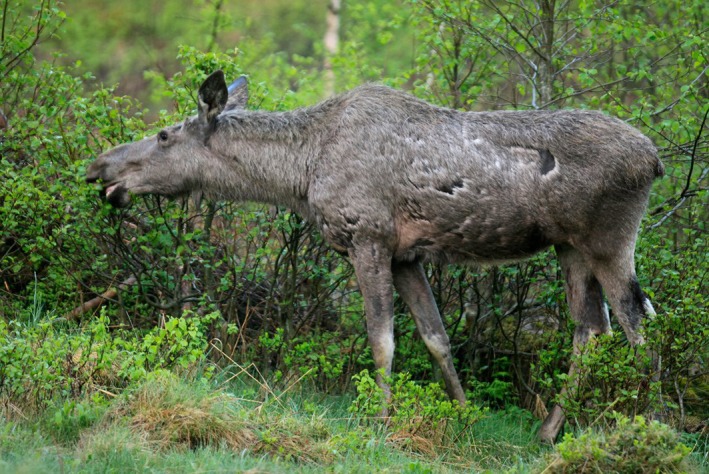
Adult moose (*Alces alces*) feeding on birches (*Betula* spp.) in early summer, southern Norway. Photo: Hallgeir B. Skjelstad

**Figure 2:**
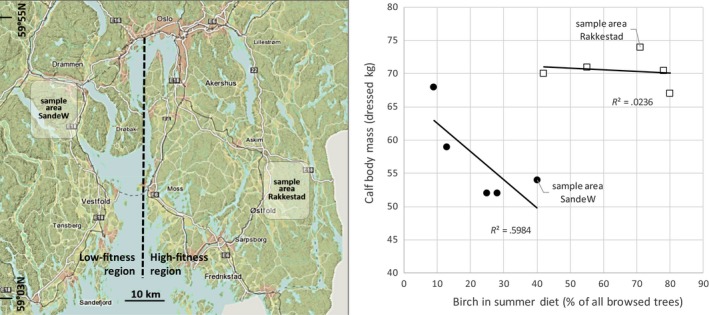
Contrasting moose fitness and utilization of a readily available food source (*Betula pubescens* Ehr.) in five study areas within two regions of southern Norway (modified from Wam et al., 2010, 2016). Birch density was high in all areas (2,470 ± 252 birches available/ha in the low‐fitness region, and 4,659 ± 311 in the high‐fitness region, and <20% of available birches were browsed in both regions). Birch in the diet was estimated from counting browse marks on woody plant species along line transects and corrected for nonwoody diet contents as found by fecal analyses (see Wam & Hjeljord, 2010a). In this paper, one sample area in each region was used to collect and analyze nutritional composition of available and used birch foliage, with the aim to explore why moose in the low‐fitness region does not utilize the readily available birch to a larger extent. Presumably, birch could be a remedy if food shortage is their culprit for higher fitness

Keeping these multiple nutritional factors and complexities in mind, in this study, we measured nutritional composition of foliage from a staple food source (*Betula pubescens* Ehr.) available to, and used by, moose (*Alces alces*) (Figure 1) in natural settings of two neighboring regions of southern Norway with contrasting animal fitness (here, indexed by body mass, which is found to capture much of the fitness variance among Fennoscandian moose populations, Tiilikainen, Solberg, Nygrén, & Pusenius, 2012). Long‐term research focus has not managed to fully explain the contrasts between the populations' food selection and demographic performance (Hagen, 1983; Hjeljord & Histøl, 1999; Wam, Histøl, Nybakken, Solberg, & Hjeljord, 2016). Although moose in both regions have access to birch in excess per capita (Wam et al., 2010), the low‐fitness population utilizes it to a noticeably lower extent than does the high‐fitness population (Figure 2). The study was initiated to explore whether the nutritional composition of the birch foliage could explain this apparent underutilization. We tested whether contents of food constituents (crude protein [CP], fiber fractions, water‐soluble carbohydrates [WSC], minerals, and plant secondary metabolites [PSM]) differed between areas, and between used and available foliage. We then used principal component analyses (PCA) to place the differences in a multidimensional framework, considering constituents in synchrony.

## 2 METHODS

### 2.1 Study area

The two sample areas used for foliage analyses in this study are situated 100 km apart in southeastern Norway (SandeW at 59°42′N, 10°7′E in the low‐fitness region and Rakkestad at 59°30′N, 11°22′E in the high‐fitness region). A fjord and densely populated areas practically eliminate exchanges of moose between the low‐ and high‐fitness regions to which the sample areas belong (Figure 2). Both regions are part of the boreal forest zone (Moen, 1999), dominated by commercially cultivated Norway spruce (*Picea abies*), with some Scots pine (*Pinus sylvestris*) on drier sites of poor soil fertility. Younger forest stages are dominated by birch (*Betula* spp.), sparsely intermixed with other deciduous species: in SandeW, birch make up 78% of the browse biomass on a typical clearcut (<20 years since logging, intermediate soil fertility), compared to 95% in Rakkestad (Wam et al., 2010). In the field layer, bilberry (*Vaccinum myrtillus*) is the most abundant forage plant in the older forest, and grasses in the younger forest. Comprehensive data on plant abundances are given in Wam and Hjeljord (2010a).

The soil fertility in the study area is intermediate, and generally a little higher in SandeW, where approximately 60% of area is classified as ≥G14 (in the H_40_ forest productivity index) compared to 40% in Rakkestad (Wam et al., 2010) (see Tveite, 1977 for details on the H_40_ index, which indicates tree height when trees are 40 years at breast height = 1.3 m). A typical clearcut on <G14 sites produces only about half as much deciduous browse as do clearcuts on the more fertile sites. Practically, all loggings are performed as clearfelling, and clearcuts are small (averaging about 1.5 ha) in a global perspective. Tops and branches are traditionally left on site to decompose, and new spruce forest is almost entirely recruited by planting. The use of herbicides, pesticides, scarification, and fertilizers is generally scarce in the area and had not been applied on the clearcuts used for foliage sampling in this study.

The climate in the study area is continental with cold winters (February norm −4.5°C in SandeW and −5.6°C in Rakkestad) and warm summers (June norm 14.9°C in SandeW and 13.7°C in Rakkestad) (Norwegian Meteorological Institute, 2013). Start of growing season (first day of the year with mean temperature >5°C) is 2 May in SandeW and 25 April in Rakkestad. Normal precipitation during June is 59 mm (SandeW) and 65 mm (Rakkestad). Norms are based on the years 1961–1990.

### 2.2 Data collection

We collected foliage for obtaining nutritional profiles between 19/06/2012 and 06/07/2012 and between 24/06/2013 and 12/07/2013, alternating between the low‐fitness and the high‐fitness region every 3rd day to avoid bias from sample date. Sites to be sampled were randomly drawn from all available clearcuts of age 5, 10, or 15 (±1 year) years since clearing (8 replicas of each), on intermediate soil fertility (defined as G14 or G17 on the H_40_ system) (*N *=* *24 clearcuts each for SandeW and Rakkestad). The same clearcuts were sampled in 2 years in order to account for potential influence of weather, which strongly influences nutrient composition (and moose selection) of browse (Bø & Hjeljord, 1991). June was colder and drier in 2012 than in 2013: mean temperature/precipitation was 12.1°C/83 mm versus 13.8°C/142 mm in SandeW, and 11.8°C/108 mm versus 13.6°C/134 mm in Rakkestad (Norwegian Meteorological Institute, 2013).

We systematically sampled foliage from *N *=* *9 pristine trees (no obvious signs of herbivory, damage, or disease) per clearcut each year. These samples represent the “available” birch foliage in our study. Sampling was systematically spread out along a fixed cross‐sectional pattern of the clearcut, starting with one tree in the center and two each in the four perpendicular directions from center (excluding the last 10 m to avoid edge effects). The aim was to spatially distribute our sampling as much as possible in order to cover the most variance in local growth conditions for birch on the clearcut and to avoid observer bias when selecting trees. We therefore kept a straight path, not yielding for hurdles like ditches or piles of logging waste. After walking the set distance, we sampled the nearest available tree. We opted to include only pristine trees when sampling for available foliage, because plant responses to herbivory attacks are so diverse (Kessler & Baldwin, 2002). One can theorize that we thereby omitted attractive foliage, and rather sampled what had been discarded by moose. However, there were thousands of birches on the clearcuts in the study area (4,659 ± 311/ha in Rakkestad, 2,470 ± 252 in SandeW, *N *=* *576 plots), and only 16 ± 2.4% (Rakkestad) and 15 ± 1.9% (SandeW) of the available trees were browsed by moose in summer (H. K. Wam, unpublished data). We therefore consider our samples to be a random selection of the available foliage, and thus a representation of its average nutritional composition on the clearcuts.

Trees with signs of current summer browsing by moose (i.e., leaf stripping) were sampled opportunistically throughout the clearcut. Upon visually detecting leaf stripping, we consistently sampled the tree closest to us (sometimes several trees in a cluster were stripped). Samples could not stem from the same cluster of trees. These samples represent the “used” foliage in our study. We assume they reflect the carte blanche choice of moose, that is, their individual nutritional composition had not (yet) been influenced by the browsing, and thus, reflect the nutritional composition that moose were seeking in this foliage. This approach is the only option when one wishes not to manipulate the foodscape. Three clearcuts in SandeW lacked browsed trees.

We defoliated each sample tree mimicking moose browsing along the 20–30 outer cm of the current year's growth of the leader shoot (including leaves and minor parts of petioles). If the leader shoot on used trees had been browsed by moose, we defoliated the neighboring shoot. Summer browsing intensity per tree was very low, with only a few shoots browsed per tree (Wam & Hjeljord, 2010b). Therefore, sampled foliage always stemmed from shoots in the central apex portion of the tree (not from the side branches, which may have a different chemical composition Hjeljord, Høvik, & Pedersen, 1990). Composite samples of available and used foliage, respectively, were combined in sealed plastic bags on site and placed in open paper traces when we returned to the field quarters in the afternoon. The foliage was then let to air‐dry inside with no exposure to sunlight. After 3–5 days, foliage had reached a constant dry weight concentration of 91.1 ± 0.13%.

### 2.3 Chemical analyses

We measured concentrations of low molecular weight phenolics directly on the air‐dried samples from 2013. The phenolic measures are stated per dry weight (mg per DW). Briefly outlined, we ground the samples, conducted four series of cold‐methanol extractions and then ran the samples through High Pressure Liquid Chromatography (HPLC, 1100 series, Agilent USA) (for more details, see Nybakken, Hörkkä, & Julkunen‐Tiitto, 2012). We quantified phenolic acids and flavonoids at 320 nm. Individual compound concentrations were calculated based on available commercial standards. In the result section, we have collected these into major groups, while the individual measures are listed in the appendix (Table A1). We analyzed condensed tannins from the HPLC‐extract (MeOH‐soluble fraction) and from the dried residue after phenolic extractions (MeOH‐insoluble fraction) with the acid butanol assay (Hagerman, 2002). We calculated these concentrations using purified condensed tannins from *Betula nana* (dwarf birch) leaves.

Prior to nutrient analyses, we estimated dry matter concentration by oven‐drying subsamples at 103°C. Because dry matter concentrations of samples already were so high from air drying (91.1 ± 0.13%), samples analyzed for nutritional contents were not additionally dried in the laboratory. All nutrient measures are stated per dry matter, that is, corrected for remaining moisture in the dried samples. We estimated CP using a thermal conductivity detector (Leco FP‐528; Leco^®^, St. Joseph, USA) and the 990.03 calculation (a standard established by the Association of Official Analytical Chemists, AOAC, 2012). We determined structural carbohydrates using filter bag techniques (Ankom Technology A200), that is, method 6 for neutral detergent fiber (NDF) (Van Soest, Robertson, & Lewis, 1991), method 5 for acid detergent fiber, and method 9 for acid detergent lignin (Daisy II Incubator, solution as in 973.18, AOAC, 2012). The contents of fiber fractions stated in the text are adjusted for residual starch and protein (i.e., aNDF), but not for residual ash (i.e., not aNDFom). We estimated WSC with a spectrophotometer (Genesys 10S Vis; Thermo Fisher Scientific, Inc., Waltham, USA), following Hall, Hoover, Jennings, and Miller Webster (1999). We analyzed contents of minerals with inductively coupled plasma spectrometry (ICP‐AES) (iCAP 6300 Radial; Thermo Fisher Scientific, Inc., Waltham, USA) after microwave digestion (EAM sec. 4.4, FDA, 2013).

### 2.4 Statistical analyses

We tested for differences in nutritional compositions using factorial analyses of variance (“lm” in R, version 2.15.3, R Core Team, 2013), with use (available, used), region (high fitness, low fitness) and year (2012, 2013) as categorical predictors (specified as factors). Homogeneity of response variances across each predictor level was checked by graphical inspection of residuals from exploratory linear fits (Zuur, Ieno, & Smith, 2007) and found adequate apart from for sodium. We therefore opted to use observations directly, with no variance‐stabilizing transformations. Each response parameter (nutrient or chemical group of secondary compounds) was tested in a separate model with the explanatory predictors (use, region, year) as fixed effects. Generalized models fitted with logit link function and binomial distribution for proportional data (McCullagh & Nelder, 1989) gave consistently the same outcome as our ordinary linear models. To facilitate direct interpretation of model output, we prefer not to transform data unless necessary. We therefore opted to present the linear models in the paper. Final models were validated by lack of patterns in residuals plotted against fitted values and QQ plots of standardized residuals (Zuur et al., 2007). To visualize how nutrient concentrations covaried, we ran principal component analyses (“prcomp” in R). Because of large differences in concentrations between nutrients (e.g., carbohydrates in the magnitude of 30% vs. trace elements in the magnitude of 3‰), we centered and scaled concentrations for each nutrient prior to the PCA (van den Berg, Hoefsloot, Westerhuis, Smilde, & van der Werf, 2006).

## 3 RESULTS

### 3.1 Nutritional composition of available and used birch foliage

**Figure 3:**
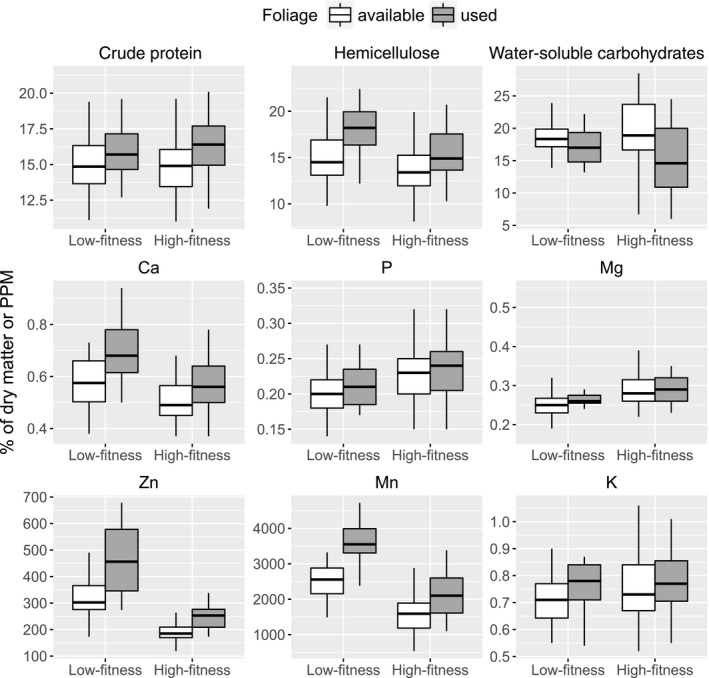
Nutritional profiles of birch foliage available to and used by moose in two Norwegian regions with contrasting animal fitness, late June to early July 2012–2013. Shown are median with 1st–3rd quartiles (boxes) and 1.5 cut‐off for min and max (whiskers) for nutrients where used foliage significantly differed from available foliage. See Table 1 for complete nutritional profiles, as well as the influence of year

Macronutrient concentrations in the available birch foliage were largely similar in the two regions (Table 1, Figure 3), with only hemicellulose and cellulose being slightly lower in the high‐fitness region (hemicellulose only in year 2013, Figure 4). Notably, there was also a wider range of available concentrations of WSC in the available foliage in the high‐fitness region, especially in 2013 (thus a significant area × year interaction). Area differences were stronger for minerals: the low‐fitness region had available birch foliage with more calcium and zinc (both more so in 2013), as well as more iron, copper, and manganese than the high‐fitness region. In contrast, birch foliage in the low‐fitness region had less phosphorous and magnesium (Mg only in 2012). According to the PCA, nutrients in the available foliage covaried in a distinct pattern: calcium, zinc, manganese, and structural carbohydrates formed one cluster, while potassium, copper, phosphorous, and CP formed another cluster, and magnesium and WSC a third cluster (Figure 5a). It was mainly the first and third of these clusters that separated the two regions in the biplots.

**Table 1:** Concentrations of nutrients (% of dry matter) and plant secondary compounds (mg per dry weight) in birch foliage available to or used^a^ by moose on boreal forest clearcuts (*N* = 48)^b^ in two Norwegian regions with contrasting animal fitness (low and high body mass). Foliage was sampled in late June to early July 2012 and 2013 (June 2012 was colder and drier than 2013). Statistical tests^c^ ran as sequential contrasting against the reference level high‐fitness region, available foliage, year 2012 (i.e., the intercept). Single coefficients must be interpreted in relation to the reference level and interaction effects. A simplified example of how to read the table: lignin concentration was 7.3 in the available birch foliage for both regions in 2012 (no influence of “area”), while it was 0.6 lower in 2013 and 1.8 lower in used than in available. However, there was a positive year × use interaction which largely outweighs these two negative influences (lignin was actually higher in used than in available foliage in 2013, see also Figure 4). We have put coefficients most central to interpreting difference between available and used in bold font

Response	Coefficients [*t*,* p*‐value]						
	α (intercept)	β_1_ (area)	β_2_ (year)	β_3_ (use)	β_1_ × β_2_	β_1_ × β_3_	β_2_ × β_3_
Crude protein (%)	15.0	n.s	n.s	**1.2 [2.8, 0.005]**	n.s	n.s	—
Neutral detergent fiber (NDF) (%)^d^	30.1	n.s	1.4 [1.5, 0.144]	−1.9 [−1.5, 0.138]	n.s	n.s	**4.7 [2.7, 0.007]**
Acid detergent fiber (ADF) (%)	16.9	n.s	−0.7 [−1.8, 0.083]	−1.3 [−2.4, 0.016]	n.s	n.s	**2.1 [3.0, 0.004]**
Lignin (%)	7.3	n.s	−0.6 [−2.5, 0.013]	−1.8 [−2.4, 0.018]	n.s	n.s	**2.0 [4.6, 0.000]**
Hemicellulose (NDF – ADF)	13.7	0.3 [0.5, 0.641]	0.1 [0.1, 0.913]	−0.6 [−0.7, 0.490]	2.5 [2.4, 0.018]	n.s	**3.5 [3.1, 0.003]**
Cellulose (ADF – lignin)	9.3	0.6 [3.0, 0.004]	n.s	n.s	n.s	n.s	n.s
Water‐soluble carbohydrates (WSC) (%)	22.3	−2.6 [−2.7, 0.007]	−8.1 [−8.6, 0.000]	−**1.8[**−**2.2, 0.027]**	5.9 [4.5, 0.000]	n.s	n.s
Calcium (Ca) (‰)	5.2	0.4 [1.4, 0.152]	−0.3 [−1.3, 0.200]	**0.9 [4.2, 0.000]**	0.9 [2.6, 0.014]	n.s	n.s
Phosphorous (P) (‰)	2.4	−0.2 [−2.6, 0.010]	−0.2 [−2.0, 0.040]	n.s	n.s	n.s	n.s
Magnesium (Mg) (‰)	3.0	−0.5 [−6.1, 0.000]	−0.2 [−2.7, 0.008]	n.s	0.4 [3.0, 0.004]	n.s	n.s
Potassium (K) (‰)	7.2	n.s	0.6 [2.4, 0.018]	**0.7 [2.1, 0.042]**	n.s	n.s	−0.6 [−1.4, 0.150]
Sodium (Na) (‰)^e^	1.0	2.6 [7.4, 0.000]	−0.4 [−1.0, 0.312]	0.1 [0.1, 0.910]	−2.8 [−5.1, 0.000]	−2.8 [−3.5, 0.000]	−0.0 [−0.0, 0.985]
Iron (Fe) (PPM)	53.8	4.4 [2.2, 0.032]	n.s	n.s	n.s	n.s	n.s
Zinc (Zn) (PPM)	190	98.2 [5.5, 0.000]	−7.5 [5.5, 0.000]	**69.1 [4.9, 0.000]**	132.1 [5.2, 0.000]	n.s	n.s
Copper (Cu) (PPM)	7.3	0.7 [4.2, 0.000]	−1.2 [−5.8, 0.000]	**0.7 [2.5, 0.013]**	n.s	n.s	−0.5 [−1.4, 0.165]
Manganese (Mn) (PPM)	1550	990 [7.1, 0.000]	n.s	**560 [3.4, 0.000]**	n.s	**494 [2.0, 0.051]**	n.s
Molybdenum (Mo) (PPM)	0.2	n.s	−0.1 [−3.3, 0.001]	n.s	n.s	n.s	n.s
MeOH‐soluble condensed tannins (mg per DW)	5.6	3.5 [5.1, 0.000]		−**1.6 [**−**2.3, 0.025]**		n.s	
MeOH‐insoluble condensed tannins (mg per DW)	31.9	−15.5 [−10.6, 0.000]		n.s		n.s	
Chlorogenic acid and derivatives (mg per DW)	1.6	2.1 [7.3, 0.000]		n.s		n.s	
Hydroxycinnamic acids (HCAs) (mg per DW)	0.9	n.s		n.s		n.s	
Myricetin glycosides (mg per DW)	1.0	n.s		−**0.2 [**−**2.0, 0.042]**		n.s	
Quercetin glycosides (mg per DW)	4.1	1.1 [2.9, 0.005]		n.s		n.s	
Kaempferol glycosides (mg per DW)	2.1	0.5 [1.7, 0.099]		0.2 [0.7, 0.471]		−0.7 [−1.7, 0.094]	
Apigenin glycosides (mg per DW)	1.0	−0.4 [−2.3, 0.026]		n.s		n.s	
Flavonoids (mg per DW)	6.4	0.9 [1.5, 0.140]		n.s		n.s	

a Available = foliage from a random sample of undamaged trees that had not (yet) been browsed by moose. Birches were available in very high densities on the clearcuts (mean 3,565 ± 282/ha across study areas), so we consider these samples to represent a cross‐section of available birch foliage (not rejected foliage). Used = foliage from trees with recent browsing marks from moose (i.e., leaf stripping).

b One municipality selected as sampling area in each region. Clearcuts were randomly drawn from all the area's clearcuts of intermediate site fertility and age 5, 10 or 15 years since clearing (balanced design). Chemical analyses on composite samples per clearcut, made from 9 ± 0.0 (available) and 3 ± 0.2 (used) trees. The same clearcuts were sampled in both years (secondary compounds only measured in 2013).

c Linear model, no transformations applied. Generalized models with logit link and binomial correction (quasi‐binomial, approximated Wald‐statistics) gave consistently the same results.

d Lignin is practically ingestible to moose, so the digestible fractions of food fiber are hemicellulose and cellulose.

e β_1_ *× *β_2_ *× *β_3_ = 2.7 (2.7, 0.009). Available foliage in the low‐fitness region in 2012 had a very large variance (and thereby a higher mean) in sodium, causing the 3‐way interaction to be significant.

**Figure 4:**
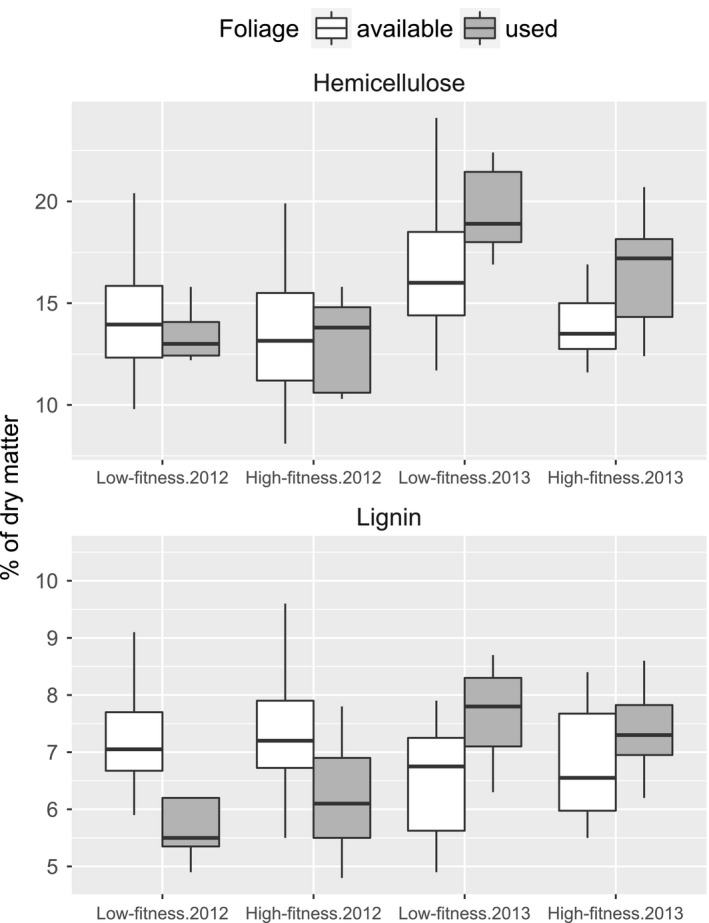
Fiber structure (hemicellulose and lignin) of birch foliage available to and used by moose in two Norwegian regions with contrasting animal fitness, late June to early July 2012–2013. Shown are median with 1st–3rd quartiles (boxes) and 1.5 cut‐off for min and max (whiskers). Note the influence of year

**Figure 5:**
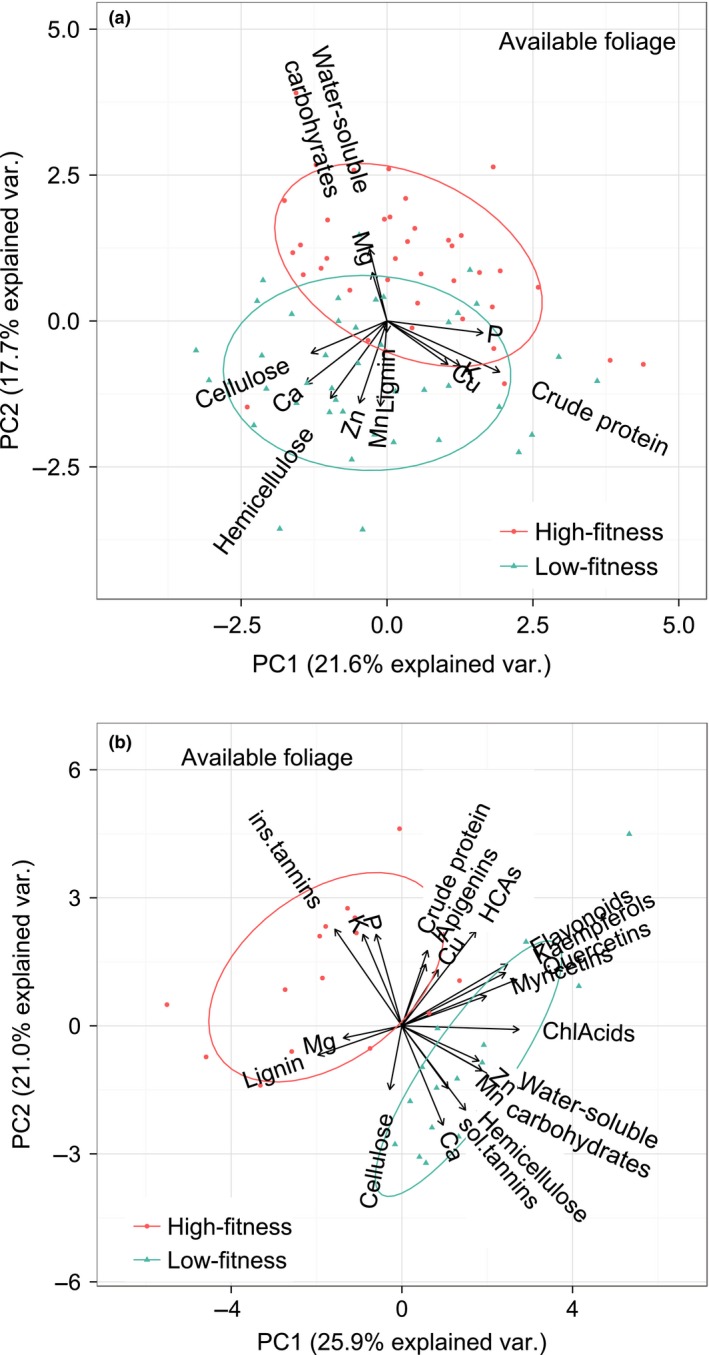
Biplots showing covariance in concentrations of (a) nutrients as well as (b) plant secondary metabolites in birch foliage available to moose in two Norwegian regions of contrasting animal fitness (high and low), late June to early July 2012–2013. Food constituents on arrows close together covary the most, and in a differing direction than other such clusters. The longer the arrow, the stronger the variance of a given nutrient follows this clustering pattern. The ellipses around observations are 2/3 confidence intervals. The less overlap between these, the larger the difference between areas. Ca, calcium; P, phosphorous; K, potassium; Zn, zinc; Mn, manganese; Cu, copper; sol.tannin, MeOH‐soluble condensed tannins; ins.tannin, MeOH‐insoluble condensed tannins; HCA, hydroxycinnamic acids; ChlAcid, chlorogenic acids

Moose showed consistent selection for a specific nutritional composition across areas, that is, there were practically no significant area × use interactions (Table 1, Figures 3 and A1). Compared to the availability, moose used foliage with more CP, calcium, zinc, manganese (particularly in the low‐fitness region), potassium, and copper, but less WSC. The selection of structural carbohydrates differed between years (Table 1): Lignin concentration in the used foliage was lower than in the available foliage in 2012, but not so in 2013 (Figure 4). In 2013, the used foliage also had more hemicellulose compared to the available foliage.

### 3.2 Contents of plant secondary metabolites

**Figure 6:**
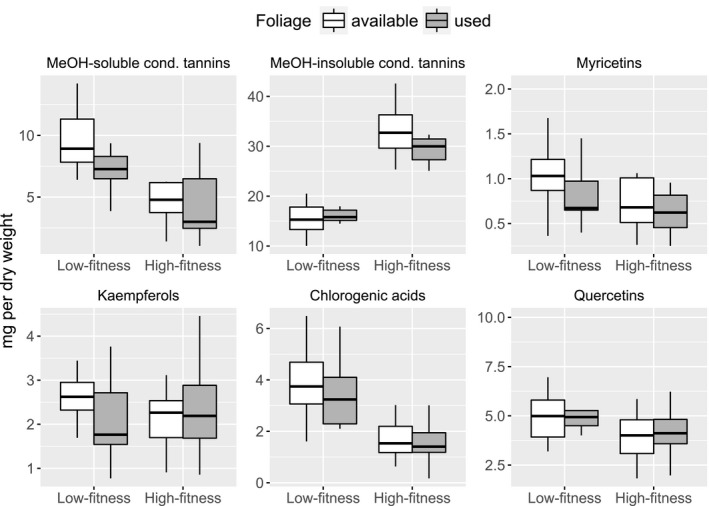
Concentrations (mg DW‐1) of plant secondary metabolites in birch foliage available to and used by moose in two Norwegian regions with contrasting animal fitness, late June to early July 2012–2013. Shown are median with 1st–3rd quartiles (boxes) and 1.5 cut‐off for min and max (whiskers) for chemical groups where used foliage significantly differed from available foliage

There were also area differences in the concentrations of plant secondary metabolites (PSM) in the birch foliage, particularly concerning MeOH‐soluble condensed tannins (Figure 6). The available foliage had less MeOH‐insoluble tannins in the low‐fitness region than it had in the high‐fitness region, but these tannins did not differ between available and used foliages. In contrast, the concentrations of MeOH‐soluble condensed tannins (and slightly also myricetins) were lower in used than in available foliage. The available foliage had almost a twice as high concentration of these tannins in the low‐fitness region than in the high‐fitness region. The low‐fitness region also had significantly more chlorogenic acids and quercetins (no difference between available and used). A principal component biplot showed clear area separation of available foliage by differences in the soluble versus insoluble condensed tannins (Figure 5b). Notably, there was also a positive covariation between the soluble condensed tannins and digestible carbohydrates (hemicellulose, cellulose, and WSC). Additional biplots indicate that moose used foliage with generally lower concentrations of PSM than available, and more so in the low‐fitness region than in the high‐fitness region (Figure A2).

## 4 DISCUSSION

Our study of moose selectivity of nutritional compositions in birch foliage in areas with contrasting fitness produced three key findings. First, the moose showed a clear selection pattern of food constituents, as the nutritional profiles of birch foliage that they used differed significantly from the birch foliage that was available to them on young clearcuts. Second, our results highlight the complex interconnections between macronutrients and micronutrients and PSM in a staple food source and their potential influences on consumers. Third, the area differences in the availability of foliage with the preferred nutritional composition offer insights into observed contrasting fitness of the distinct moose populations living there. Below, we try to integrate these key findings. It is important not to generalize the specific selection pattern of food constituents in our study (peak of growing season) to other times of the year, because this is likely to differ with season (e.g., Stolter, Ball, & Julkunen‐Tiitto, 2013; Tixier et al., 1997).

### 4.1 Selection of food constituents: A challenging balancing act

Irrespectively of region, the moose in our study consistently selected birch foliage with higher concentration of CP and several minerals (most strongly Ca, Zn, and Mn) than in the average birch foliage that was available to them. Because of covariation with other food constituents, we must not interpret the positive single statistical coefficients as intended selection for a given food constituent (Table 1), but rather look at them in synchrony. A few previous studies on moose selection of plant material within specific plant species have also shown a correlation between food selection and covarying contents of protein and minerals (e.g., Danell, Niemela, Varvikko, & Vuorisalo, 1991; Faber & Lavsund, 1999; Thompson, McQueen, Reichardt, Trenholm, & Curran, 1989). Thompson et al. (1989), for example, concluded that moose selected for stands of balsam fir which had protein contents that met their requirement. Because protein comprises a much larger part of animal diet than do minerals, it is easy to overemphasize by default the role of protein. However, perhaps an animal uses a particular food source to adjust the overall intake of other food constituents. A case in point, the “selection for” higher Ca by moose in our study may actually reflect a need for Zn, traded against the costs of accompanying excess Ca (see below).

A selection for food with higher protein contents was expected in our study because large herbivores normally can meet their protein needs only during plant growing season (Mattson, 1980; Parker et al., 2009). Our study was conducted at times of peak contents of CP in browse (e.g., Leslie, Starkey, & Vavra, 1984; Marshal, Krausman, & Bleich, 2005). Yet, whether protein contents of food actually contribute to drive food selection depends on its scarcity relative to animal needs (see, e.g., Beck, Flinders, Nelson, & Clyde, 1996; Zweifel‐Schielly, Kreuzer, Ewald, & Suter, 2009; Dostaler, Ouellet, Therrien, & Cote, 2011 compared to, e.g., Tixier et al., 1997; Gillingham, Parker, & Hanley, 2001; Zweifel‐Schielly et al., 2012). Moose require roughly 6%–8% CP of dry matter intake for maintenance (Schwartz, Regelin, & Franzmann, 1987), but up to 25% for reproduction and growth (review across cervids; Dryden, 2011). The birch foliage in our study had about 15% CP, that is, sufficient for maintenance, but possibly deficit for production.

Protein is not only a source of amino acids for cell renewal but also energy. This is pertinent to the question of whether a search for protein ever drives the food selection of an animal (Felton et al., 2009): is a selection for food with more protein due to a need for energy or for amino acids? In our study, protein was higher in used than available foliage, while other sources of easily digested energy were not (especially WSC, Figure 3). This indicates that amino acids were of greater physiological importance to moose than was energy when it foraged on birch foliage. To fully disentangle these two attributions, we would have to look at the complete food intake (Felton et al., 2016), taking into account all the potential covariations of importance as indicated by the biplots in our study (Figure 5).

The moose in our study actually used birch foliage with contents of WSC being lower than in the available birch foliage. Notably, the within‐year variation of WSC in available foliage was lower in the low‐fitness region than in the high‐fitness region, offering moose less of a choice. WSC have seldom been studied in relation to the diet of large herbivores in natural settings (but see Beck et al., 1996; Faber & Lavsund, 1999; Tixier et al., 1997), and it seems premature to routinely ignore it. WSC are one of the highly fermentable sources of energy that is assumed to increase palatability for most animal species (e.g., Jones & Roberts, 1991). However, it could be that there are interactions with other food constituents that wildlife research is not yet aware of. One such may be positive covariation with soluble condensed tannins (Figure 5b).

In contrast to protein and WSC, the moose in our study appeared to be quite flexible on the fiber structure when selecting birch foliage, with hemicellulose in used foliage being similar as in available foliage in 1 year (2012) and higher in the other year (2013). This is a fine reminder to ecologists that weather conditions produce interannual variation in nutritional compositions (e.g., Vázquez‐de‐Aldana, García‐Ciudad, & García‐Criado, 2008), and subsequently in animals' food selection. Interestingly, the foliage used also had higher lignin concentrations in 2013 than in 2012. Because lignin is practically indigestible to ruminants (Van Soest, 1994), it is generally expected that they select for food with lower lignin concentrations. Possibly, the moose had to “accept” birch foliage with more lignin in 2013 because the benefits from other food constituents outweigh the reduced digestibility from lignin.

No other nutrients than fiber had a significant year × use interaction in our study, despite the fact that practically all the mineral concentrations in the available birch foliage varied with year (and area). This indicates high importance of mineral compositions to the animals. Ceacero, Landete‐Castillejos, Garcia, Estevez, and Gallego (2010) have documented that cervid individuals are indeed able to adjust their mineral intake according to the nutritional needs. Different minerals are absorbed and function in strong interaction (nutrient stoichiometry, Elser et al., 2000), and also in relation to macronutrients. The importance of balancing the intake of various minerals is well‐known within livestock research (Reece, Erickson, Goff, & Uemura, 2015) but has received little attention in wildlife literature.

Findings in our study that stand out in relation to minerals mainly pertain to Ca, Zn, Cu, and Mn. Although P and Mg were of similar concentrations in used and available birch foliage, it is also worth noting the area difference in their ratios to Ca. The ratio between Ca, P, and Mg is crucial for calcium homeostasis which is one of the most sensitive homeostasis in the body (Arnaud, 1983). In large herbivores, Ca in the blood must be maintained within the narrow range of 1.00–1.25 mmol/L (NRC, 2001). The Ca ratios to other minerals need to be narrowly balanced because minerals affect each other's absorption in the animal body by forming insoluble complexes (Spears, 2003). The Ca:P:Mg mineral ratios are of particular importance for moose during the growing season (i.e., our time of study), which is also the period of intensive bone (in juveniles) and antler (in males) growth. A Ca:P ratio in cervid antlers of 2:1 is highly consistent across species (Dryden, 2016). The low‐fitness region had available birch foliage with more Ca, and simultaneously a little less P and Mg (ratio Ca:P:Mg = 2.8:1:1.2) than the high‐fitness region (ratio 2.2:1:1.3). Particularly, the former ratio is not within recommendations for large ruminants (NRC, 2001), where Ca intake should preferentially stay within 1–1.5 times the P and Mg intake (lower ratios for maintenance than for bone production). The moose in our study did not select birch foliage in line with NRC recommendations, however, as the ratios were even higher in used than in available foliage (ratio used 3.3:1:1.2 for low fitness, and 2.4:1:1.2 for high fitness). This may be a result of constraints in selection options rather than overall nutritional preferences, as the minerals (as are all food constituents) are only available to the animals as intricate complexes in “food packages.” Possibly the moose could balance these mineral ratios by adjusting the intake of other food items in the diet.

As expected from the mineral‐binding properties of fiber (Schwartz, Regelin, Franzmann, & Hubbert, 1987; Whitehead, Goulden, & Hartley, 1985), several of the minerals in our study covaried with hemicellulose (Ca, Zn, and Mn, Figure 5a). Copper on the other hand, covaried with protein, as well as P and K. The protein‐P‐K covariation (in an opposite direction of Ca) has previously been demonstrated in cervid food (e.g., Vangilder et al., 1982). These two major interacting complexes put limitations on the moose' option to compose a nutritionally balanced diet from birch. If the moose are in need of protein, P, Cu, or K and use birch to balance their dietary intake, they will have to also accept lower contents of hemicellulose and different concentrations of Ca, Zn, and Mg. Likewise, if the moose are in need of Zn, it may have to accept surplus Ca or Mn, which comprises yet another important trade‐off.

An excess of Ca intake is known to exacerbate a deficiency of Zn (and a range of other minerals, Spears, 2003). Zn is part of a vast array of enzymes involved in especially amino acid synthesis and cell replication. A deficit therefore typically affects animal tissue growth and reproduction (e.g., Enjalbert, Lebreton, & Salat, 2006), and subsequently, causes low body mass. Ohlson and Staaland (2001) found that Zn was one of the minerals that were of higher concentrations in birch than in most other moose foraging plants in southern Norway (and in line with Zn concentrations in our study, i.e., approximately 200 PPM compared to <50 PPM in other plants, see also Suttle, 2010). Birch may therefore be of special interest to moose as a source of Zn.

In neither region did the moose show a strong general avoidance of PSM. This is in line with previous studies of moose and PSM avoidance for summer foliage (Stolter et al., 2013), as opposed to for winter twigs (Stolter, 2008). The biplots in the appendix (Figure A2) indicate that moose selected foliage with overall less PSM, but in the constituent‐specific models (Table 1), only MeOH‐soluble condensed tannins (and to a lesser extent myricetins) had a significantly different concentration of available and used. Very little is known about cervid food selection and myricetins (often considered a beneficial antioxidant to humans, Williamson & Manach, 2005). A previous study on moose showed a positive relationship between food use and myricetins contents in summer (Stolter et al., 2013). Possibly, the negative relationship in our study stems from covariation with the soluble tannins (Figure 5b, A2).

The classical studies of Robbins, Hanley, et al. (1987) and Robbins, Mole, Hagerman, and Hanley (1987) found that condensed tannins reduce protein digestibility for ruminants. Subsequent studies have confirmed this (e.g., Hagerman & Robbins, 1993; Jones, Rude, et al., 2010; Spalinger, Collins, Hanley, Casara, & Carnahan, 2010) but also added nuances to the relationship: tannins may actually be beneficial at some concentrations (Clauss et al., 2003; Min, Barry, Attwood, & McNabb, 2003), and its influence on intake may differ with season (Chapman, Bork, Donkor, & Hudson, 2010) or concentrations of other nutrients in the diet (Villalba & Provenza, 2005). From the above references on moose and tannins, it seems that for each 1% increase in condensed tannin concentration, the digestibility of CP in shrubs or browse foliage is reduced by 2.5%. Applying these numbers to our study, soluble condensed tannins in the available foliage may reduce protein digestibility by about 22% in the low‐fitness region, and by about 12% in the high‐fitness region. This falls well in line with Spalinger et al. (2010), who found that the reduction was 38% across a range of natural browse for moose in Alaska. This could be a substantial loss if protein is scarce. Notably, the moose were able to select foliage with 3 times lower concentrations of soluble condensed tannins in the high‐fitness region compared to the low‐fitness region. The area differences in actual effects of tannins may be even more skewed, as the tannin: protein ratio can determine whether insoluble tannin/protein complexes will form (Hagerman & Robbins, 1987). A follow‐up of our study would be to conduct in vivo digestibility trials with the birch foliage.

### 4.2 From food selection to fitness, the next steps

Ultimately, research on nutritional ecology is directed to understand higher level ecosystem interactions, typically along the pathway of animal fitness (DeGabriel et al., 2014). Access to food that better match the preferred nutritional composition is beneficial to wide‐roaming animals such as moose for two reasons. One is the improved nutrient absorption discussed throughout the previous section. The other is the reduced energetic costs of obtaining the nutrients in the landscape (locomotion Fryxell, 1991; or predator vigilance Christianson & Creel, 2010). Our study highlights the need to take into account that the realized value of a given food source to an animal may be site‐specific. In our study, the most abundant food available to moose had a less optimal nutritional composition in the low‐fitness region compared to in the high‐fitness region, but still moose in both areas selected for the same nutritional composition of this food source. Such fastidiousness limits the amount of available food that is acceptable to the animal and has bearings on our understanding and application of the concept of carrying capacity. To better find out how, we encourage researchers to conduct food selection studies from a multidimensional viewpoint, by assessing food constituents in synchrony. This can clarify the functional roles of different constituents and the animals' nutritional priorities in times of scarcity. Another key element for future studies that is currently lacking is the bioactivity of specific PSM in the animal body, that is how they impact nutrition and subsequently, animal physiology. With such developments, we would gain a further understanding of the complex trade‐offs involved in the foraging decisions made by wide‐roaming and long‐living herbivores.

## CONFLICT OF INTEREST

The authors declare they have no conflict of interests regarding this study.
