# Supplementary material for: Moose selecting for specific nutritional composition of birch places limits on food acceptability
Source: Ecol Evol. 2017 Dec 20;8(2):1117–30. doi: 10.1002/ece3.3715 (PMC5773297; doi:10.1002/ece3.3715)
Supplement: Supplementary file 1 [file ECE3-8-1117-s001.pdf]

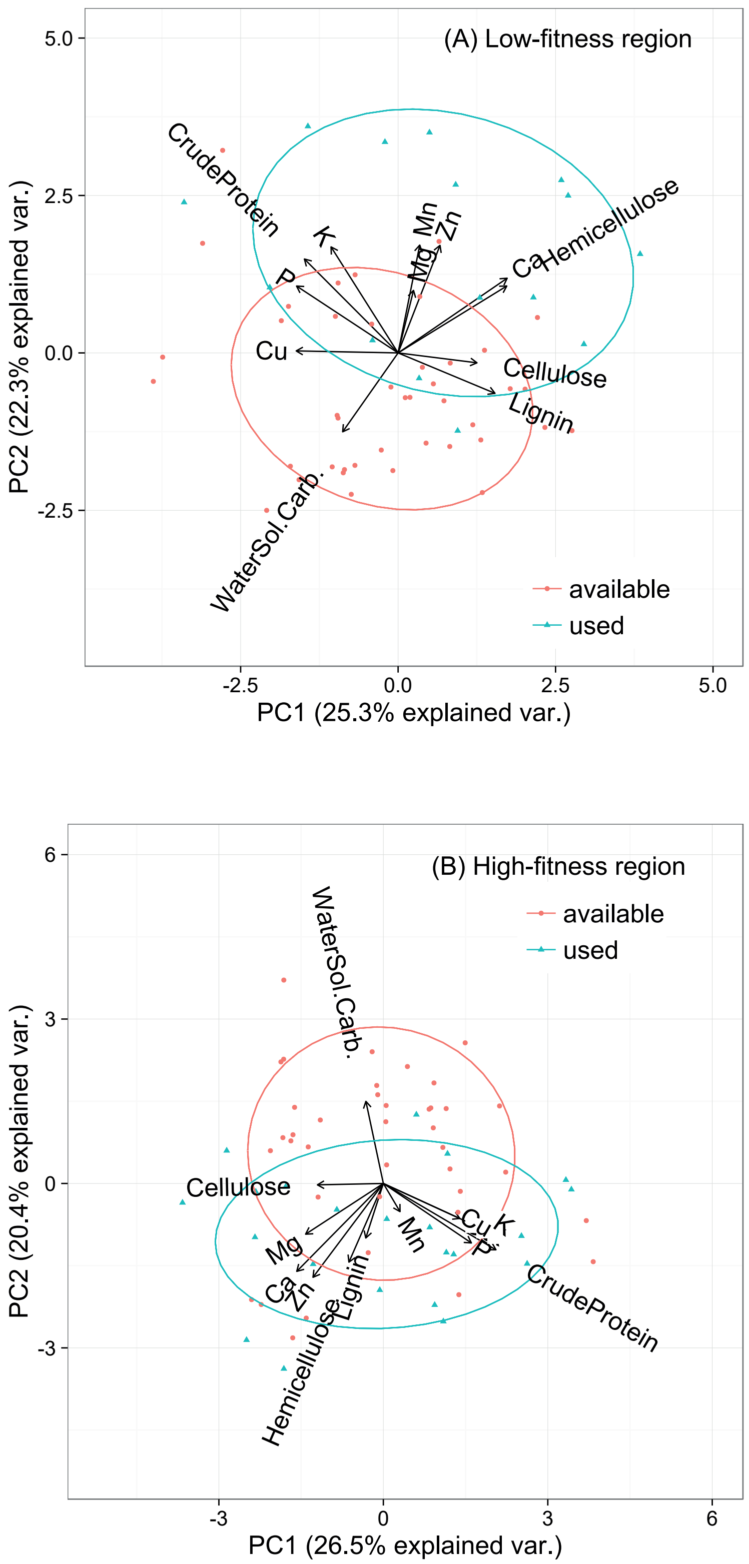

Fig. A1 *Bi-plots showing co-variance in concentrations of nutrients in birch foliage available to and used by moose in two Norwegian regions of contrasting animal fitness* ***(A)*** *low and* ***(B)*** *high, summer 2012-2013. Food constituents on arrows close together covary the most, and in a differing direction than other such clusters. The longer the arrow, the stronger the variance of a given nutrient follows this clustering pattern. The ellipses around observations are 2/3 confidence intervals. The less overlap between these, the larger the difference between available and used foliage. Abbrevations = calcium (Ca), phosphorous (P), potassium (K), zinc (Zn), manganese (Mn) and copper (Cu).*

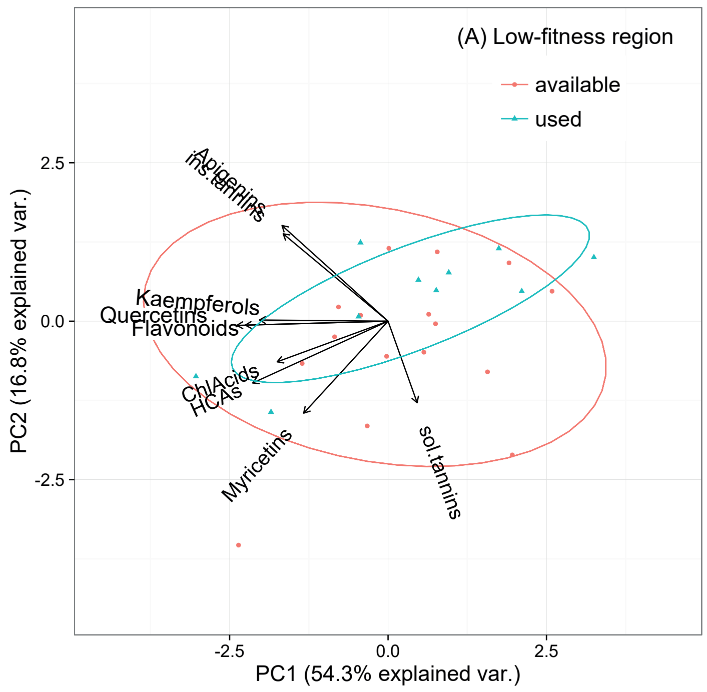

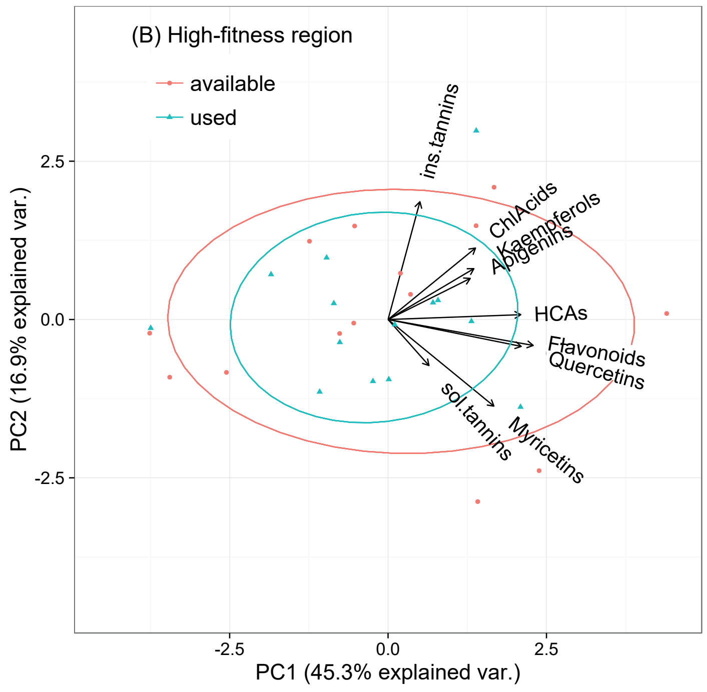

Fig. A2 *Bi-plots showing co-variance in concentrations of plant secondary metabolites in birch foliage available to and used by moose in two Norwegian regions of contrasting animal fitness* ***(A)*** *low and* ***(B)*** *high, summer 2012-2013. Food constituents on arrows close together covary the most, and in a differing direction than other such clusters. The longer the arrow, the stronger the variance of a given nutrient follows this clustering pattern. The ellipses around observations are 2/3 confidence intervals. The less overlap between these, the larger the difference between available and used foliage. Abbrevations = MeOH-soluble condensed tannins (sol.tannin), MeOH-insoluble condensed tannins (ins.tannin), hydroxycinnamic acids (HCAs), Chlorogenic acids (ChlAcids).*

Table A1. Concentrations (mg g^{-1} DW) of phenolic compounds (mean ± 1 S.E.) in birch foliage available to or used [a] by moose on boreal forest clearcuts (*N* = 48[b]) in two Norwegian regions with contrasting animal fitness (low, high), late June to early July 2013.

	High-fitness region		Low-fitness region	
	Used	Available	Used	Available
Chlorogenic acid and derivatives	1.59 ± 0.21	1.68 ± 0.18	4.05 ± 0.60	3.71 ± 0.32
Hydroxycinnamic acid (HCA) der 1	0.06 ± 0.01	0.07 ± 0.01	0.15 ± 0.02	0.13 ± 0.01
Hydroxycinnamic acid (HCA) der 2	0.41 ± 0.05	0.47 ± 0.06	0.32 ± 0.04	0.32 ± 0.05
Hydroxycinnamic acid (HCA) der 3	0.07 ± 0.01	0.07 ± 0.01	0.07 ± 0.01	0.08 ± 0.01
Hydroxycinnamic acid (HCA) der 4	0.06 ± 0.001	0.05 ± 0.01	0.04 ± 0.01	0.04 ± 0.01
Hydroxycinnamic acid (HCA) der 5	0.04 ± 0.001	0.05 ± 0.01	0.05 ± 0.01	0.05 ± 0.01
Hydroxycinnamic acid (HCA) der 6	0.02 ± 0.003	0.03 ± 0.01	0.02 ± 0.003	0.02 ± 0.003
Hydroxycinnamic acid (HCA) der 7	0.03 ± 0.004	0.02 ± 0.01	-	0.02 ± 0.01
Hydroxycinnamic acid (HCA) der 8	0.02 ± 0.002	0.04 ± 0.01	0.01 ± 0.001	0.02 ± 0.003
Hydroxycinnamic acid (HCA) der 9	0.02 ± 0.002	0.03 ± 0.01	0.01 ± 0.003	0.01 ± 0.003
Hydroxycinnamic acid (HCA) der 10	0.04 ± 0.003	0.05 ± 0.01	0.03 ± 0.005	0.03 ± 0.003
Hydroxycinnamic acid (HCA) der 11	0.02 ± 0.002	0.03 ± 0.01	0.03 ± 0.01	0.03 ± 0.007
**Sum Phenolic acids**	2.41 ± 0.22	2.60 ± 0.27	4.77 ± 0.68	4.53 ± 0.38
myricetin3galactoside+glucoronide	0.55 ± 0.06	0.71 ± 0.12	0.74 ± 0.10	1.01 ± 0.14
myricetin glycoside 1	0.13 ± 0.02	0.18 ± 0.04	0.09 ± 0.02	0.23 ± 0.08
**Sum myricetin glycosides**	0.68 ± 0.08	0.89 ± 0.15	0.83 ± 0.11	1.23 ± 0.21
quercetin3galactoside	1.12 ± 0.22	0.91 ± 0.16	1.08 ± 0.14	1.11 ± 0.11
quercetin3glucoside	2.03 ± 0.17	1.95 ± 0.18	3.01 ± 0.29	2.80 ± 0.23
quercetin3glucuronide	0.30 ± 0.07	0.22 ± 0.04	0.16 ± 0.02	0.38 ± 0.06
querc3arabinofuranoside	0.41 ± 0.04	0.49 ± 0.20	0.71 ± 0.05	0.77 ± 0.07
quercetin glycoside 1	0.10 ± 0.02	0.17 ± 0.04	0.38 ± 0.06	0.37 ± 0.08
quercetin glycoside 2	0.32 ± 0.04	0.24 ± 0.03	0.12 ± 0.02	0.14 ± 0.02
**Sum quercetin glycosides**	4.14 ± 0.28	4.01 ± 0.31	5.14 ± 0.44	5.14 ± 0.41
kaempferol3glucoside	0.40 ± 0.04	0.36 ± 0.03	0.39 ± 0.08	0.48 ± 0.04
kaempferol3glucuronide	0.36 ± 0.03	0.48 ± 0.05	0.84 ± 0.09	0.69 ± 0.05
kaempferl3rhamnoside	0.23 ± 0.04	0.37 ± 0.07	0.21 ± 0.04	0.68 ± 0.09
kaempferol glycoside 1	0.59 ± 0.10	0.71 ± 0.11	0.65 ± 0.13	0.72 ± 0.05
kaempferol glycoside 2	0.63 ± 0.09	0.79 ± 0.14	0.51 ± 0.07	0.86 ± 0.11
Kaempferol glycoside 3	0.79 ± 0.19	0.53 ± 0.08	-	0.27 ± 0.05
**Sum kaempferol glycosides**	2.34 ± 0.30	2.12 ± 0.17	2.08 ± 0.25	2.75 ± 0.21
apigenin glycoside 1	0.12 ± 0.01	0.14 ± 0.02	0.20 ± 0.03	0.20 ± 0.02
apigenin glycoside 2	0.79 ± 0.09	1.12 ± 0.13	0.12 ± 0.03	0.83 ± 0.05
**Sum apigenin glycosides**	0.92 ± 0.10	1.09 ± 0.17	0.57 ± 0.11	0.72 ± 0.18
naringenin	0.35 ± 0.04	0.30 ± 0.04	0.12 ± 0.03	0.22 ± 0.05
eriodictyol	0.17 ± 0.03	0.17 ± 0.02	0.33 ± 0.06	0.26 ± 0.06
luteolin der	0.06 ± 0.01	0.05 ± 0.01	0.10 ± 0.02	0.06 ± 0.02
**Sum flavonoids**	6.31 ± 0.36	6.52 ± 0.55	6.99 ± 0.60	7.51 ± 0.70
**Sum low molecular phenolics**	8.72 ± 0.49	9.12 ± 0.78	11.76 ± 1.24	12.04 ± 0.99

[a] Available = foliage from a random sample of undamaged trees that had not (yet) been browsed by moose. Birches were available in very high densities on the clearcuts (mean 3 565 ± 282/ ha across study areas), so we consider these samples to represent a cross-section of available birch foliage (not rejected foliage). Used = foliage from trees with recent browsing marks from moose (i.e. leaf stripping)

[b] One municipality selected as sampling area in each region. Clearcuts were randomly drawn from all the area's clearcuts of intermediate site fertility and age 5, 10 or 15 years since clearing (balanced design). Chemical analyses on composite samples per clearcut, made from 9 ± 0.0 (available) and 3 ± 0.2 (used) trees.
